# Phylogenomic Characterization of Ranavirus Isolated from Wild Smallmouth Bass (*Micropterus dolomieu*)

**DOI:** 10.3390/v16050715

**Published:** 2024-04-30

**Authors:** Hannah Quail, Pedro H. O. Viadanna, Jordan A. Vann, Hui-Min Hsu, Andrea Pohly, Willow Smith, Scott Hansen, Nicole Nietlisbach, Danielle Godard, Thomas B. Waltzek, Kuttichantran Subramaniam

**Affiliations:** 1 Department of Infectious Diseases and Immunology, College of Veterinary Medicine, University of Florida, Gainesville, FL 32611, USA; hquail@ufl.edu (H.Q.); pedroh1986@gmail.com (P.H.O.V.); jvann@ufl.edu (J.A.V.); tomwaltzek@gmail.com (T.B.W.); 2 Emerging Pathogens Institute, University of Florida, Gainesville, FL 32610, USA; 3 Wisconsin Veterinary Diagnostic Laboratory, University of Wisconsin-Madison, Madison, WI 53706, USA; hui-min.hsu@wvdl.wisc.edu (H.-M.H.); andrea.pohly@wvdl.wisc.edu (A.P.); 4 Wisconsin Department of Natural Resources, Bureau of Fisheries Management, 2801 Progress Road, Madison, WI 53716, USA; willow.smith@wisconsin.gov (W.S.); scott.hansen@wisconsin.gov (S.H.); nicole.nietlisbach@wisconsin.gov (N.N.); danielle.godard@wisconsin.gov (D.G.)

**Keywords:** largemouth bass virus, iridovirus, ranavirus, smallmouth bass

## Abstract

In September 2021, 14 smallmouth bass (SMB; *Micropterus dolomieu)* with skin lesions were collected from Green Bay waters of Lake Michigan and submitted for diagnostic evaluation. All the skin samples tested positive for largemouth bass virus (LMBV) by conventional PCR. The complete genome of the LMBV (99,328 bp) isolated from a homogenized skin sample was determined using an Illumina MiSeq sequencer. A maximum likelihood (ML) phylogenetic analysis based on the 21 core iridovirus genes supported the LMBV isolated from SMB (LMBV-WVL21117) as a member of the species *Santee-Cooper ranavirus*. Pairwise nucleotide comparison of the major capsid protein (MCP) gene showed that LMBV-WVL21117 is identical to other LMBV reported from the United States and nearly identical to doctor fish virus and guppy virus 6 (99.2%) from Southeast Asia, as well as LMBV isolates from China and Thailand (99.1%). In addition, ML phylogenetic analysis based on the MCP gene suggests three genotypes of LMBV separated by region: genotype one from the United States, genotype two from Southeast Asia, and genotype three from China and Thailand. Additional research is needed to understand the prevalence and genetic diversity of LMBV strains circulating in wild and managed fish populations from different regions.

## 1. Introduction

Ranaviruses are globally emerging pathogens that infect a broad host range spanning 52 families and 175 species of ectothermic vertebrates (i.e., amphibians, fish, reptiles) across six continents [1]. Members of the genus *Ranavirus* (family *Iridoviridae*; subfamily *Alphairidovirinae*) are large viruses with a 99–140 kilobase pair double-stranded DNA genome enclosed within an enveloped, icosahedral nucleocapsid ranging 150–200 nm in diameter [2]. There are seven species listed within the genus *Ranavirus,* including *Ambystoma tigrinum virus*, *Common midwife toad virus*, *Epizootic haematopoietic necrosis virus*, *European North Atlantic ranavirus*, *Frog virus 3*, *Santee-Cooper ranavirus*, and *Singapore grouper iridovirus* [3].

Largemouth bass virus (LMBV) was categorized as a member of the species *Santee-Cooper ranavirus* based on the phylogenetic analyses of the major capsid protein (MCP) and DNA methyltransferase genes [4]. The doctor fish virus (DFV) and guppy virus 6 (GV6) are also members of the species *Santee-Cooper ranavirus* and are, therefore, closely related to LMBV [2,4,5]. LMBV has been associated with several mortality events across the United States since its identification in 1991 [6]. The first report of LMBV occurred in Lake Wier, Florida, after a decline in the largemouth bass (LMB; *Micropterus salmoides*) population prompted an investigation [6]. The isolation of this virus was an incidental finding, as the examined fish were apparently healthy. The first recorded mortality event involving clinical disease occurred in South Carolina at the Santee-Cooper Reservoir in 1995, where approximately 1000 LMB died [7]. The virus has since spread to other bodies of water along the East Coast, Midwest, and Western United States [1]. 

Largemouth bass are native to North America, serving as apex predators in lakes and rivers. Considered a keystone species, LMB control the population dynamics of prey fish within their habitats [8]. In addition to their ecological relevance, LMB are of significant economic importance worldwide. Largemouth bass aquaculture was introduced to China in the 1980’s and has since become one of the major freshwater species cultured, producing 37,500 kg/ha in Guangdong province alone [9]. Furthermore, LMB are popular trophy fish in North America, generating $1.25 billion in Florida in 2016 [10]. To support the demand in the United States, wild broodfish are collected and cultured in stock reservoirs. In 1999, LMB hatcheries in 10 states were surveyed, and subclinical infections of LMBV were found in five out of fifteen facilities [11]. Sample sizes from each hatchery varied, so determining the true and current prevalence of the disease would require further investigation. Nevertheless, the findings of the aforementioned study imply that LMBV could be unknowingly spread to naïve bodies of water through restocking efforts. In contrast, LMBV has resulted in high mortality of LMB in aquaculture facilities in Guangdong, China, causing serious economic losses [12].

Although the virulence of LMBV appears variable, infected LMB typically exhibit abnormal swimming behavior due to over-inflation of the swim bladder [13]. The disease has also been associated with coelomitis, exophthalmia, organomegaly, superficial necrosis of the internal organs, and ulcerative skin lesions [13,14]. Experimental studies have shown that fish are susceptible to infection horizontally through the water column or through ingestion of contaminated prey items [15,16]. Environmental influences, such as low dissolved oxygen in warm water or stress from fishing pressure during the summer, have been hypothesized as potential factors contributing to LMBV epizootics, but the definitive cause is unknown [1,17]. In an experimental setting, exposure to LMBV through immersion and water temperatures of 28 °C resulted in the mortality of 50% of the smallmouth bass (SMB; *Micropterus dolomieu*) population [14]. Although the pathogenesis of LMBV is not well understood, the detection of LMBV in the cutaneous mucus of infected fish and experimental infection through immersion suggests the potential involvement of the skin as a portal of entry and/or in viral shedding [14,16]. In addition to LMB, LMBV has also been isolated from a wide range of freshwater fishes (six families, 17 species), including SMB [1].

Similar to LMB, SMB are important aquatic predators that control populations of prey fish and insects within their habitat [18]. With the ability to tolerate warmer water temperatures, SMB maintain shoreline populations after other predators move into deeper water [18]. Since 2005, LMBV has been isolated from increasing numbers of moribund SMB in Pennsylvania and Chesapeake Bay watersheds [14,19]. Disease in this species is not as consistent as in LMB but causes similar clinical signs, such as coelomitis and ulcerative skin lesions [14]. Variability in the severity of disease in SMB populations may be due to factors such as environmental conditions of different geographic regions, virus strain, host age, immune status, and species.

In September of 2021, 14 wild SMB with ulcerative skin lesions were collected from Green Bay waters of Lake Michigan near the shoreline of Door County, Wisconsin, and submitted to the Wildlife and Aquatic Disease Laboratory at the University of Florida for diagnostic evaluation. All the skin samples tested positive for LMBV when using conventional PCR. Despite the repeated detection of LMBV in SMB, no genomic sequencing or characterization efforts have been conducted to elucidate viral taxonomy and evolution, host range, or interspecies transmission events. In the current study, we report the clinical findings from the infected SMB, ancillary diagnostics, isolation of LMBV from a homogenized skin sample, and phylogenomic analysis of this virus based on the 21 core iridovirus genes.

## 2. Materials and Methods

### 2.1. Sample Collection

After receiving reports from the public concerning fish observed with skin lesions, 14 SMB with skin lesions were collected from the waters along the Green Bay shoreline of Door County, WI, by the Wisconsin Department of Natural Resources (Madison, WI, USA) in September of 2021. Fish were caught by angling and were then humanely euthanized using 1 g/L Syncaine (Syndel, Ferndale, WA, USA) buffered with two parts of sodium bicarbonate. Ten of the fourteen fish received full necropsies with sample collection. Portions of the skin and kidney were processed in the field for bacteriology. Each sample was cultured on three bacteriology medium plates and incubated under different temperatures: a tryptic soy agar (TSA) plate at 22 °C, a Hsu-Shotts (HS) plate at 15 °C, and a HS plate at 22 °C [20]. The resulting colonies were then submitted to the Wisconsin Veterinary Diagnostic Laboratory at the University of Wisconsin–Madison (Madison, WI, USA) for bacterial identification. Separate skin, kidney, liver, spleen, and swim bladder tissue from 10 fish were placed in 10% neutral buffered formalin (Fisherbrand, Waltham, MA, USA) and sent to the abovementioned laboratory for histopathology. Additional skin samples from the ulcerative lesions, as well as pooled spleen, liver, and kidney samples, were collected from all 14 fish and stored in separate sterile Whirl-Pak^®^ bags. These samples were then placed on ice and shipped overnight to the Wildlife and Aquatic Disease Laboratory at the University of Florida (Gainesville, FL, USA). 

### 2.2. Histopathology

Formalin-fixed tissues (skin, kidney, liver, spleen, and swim bladder) were routinely processed and embedded in paraffin blocks. Then, 5 μm sections were cut, placed on glass slides, and stained with hematoxylin and eosin (H&E) for examination by using light microscopy.

### 2.3. Nucleic Acid Extraction and PCR Amplification

The DNA from the skin and pooled internal tissues (spleen, liver, and kidney) were separately extracted from each of the 14 fish using the AllPrep^®^ DNA/RNA Mini kit (Qiagen, Hilden, Germany) according to the manufacturer’s instructions. The concentration of the extracted DNA was measured fluorometrically with a Qubit 3.0 Fluorometer using a dsDNA BR assay (Invitrogen, Waltham, MA, USA). A LMBV conventional polymerase chain reaction (PCR) assay targeting a portion of the MCP gene [21] was completed in 30 µL reaction mixtures composed of 17.6 µL water, 3 µL of 10× buffer solution, 1.2 µL of 50 mM MgCl_2_, 0.6 µL of 10 mM dNTP mix, 1.5 µL of each 20 mM primer (LMBV288F [5′-GCGGCCAACCAGTTTAACGCAA-3′] and LMBV535R [5′-AGGACCCTAGCTCCTGCTTGAT-3′]), 0.15 µL Platinum *Taq* DNA polymerase (Invitrogen, Waltham, MA, USA), and 4.5 µL of the DNA template up to 100 ng per reaction. The amplification was conducted using a SimpliAmp Thermal Cycler (Applied Biosystems, Waltham, MA, USA) under the following parameters: 94 °C for 5 min, succeeded by 30 cycles at 94 °C for 30 s, 60 °C for 30 s, 72 °C for 30 s, and final elongation at 72 °C for 5 min. The resulting PCR products were analyzed by using electrophoresis on a 1% agarose gel stained with ethidium bromide. An amplified product (248 bp) with the brightest band was purified using a QIAquick PCR purification kit (Qiagen, Hilden, Germany), quantified using a Qubit 3.0 fluorometer, and submitted for Sanger sequencing at Functional Bioscience, Inc. (Madison, WI, USA).

### 2.4. Virus Isolation

Virus isolation was attempted on *Epithelioma papulosum cyprini* cells (EPC) [22] incubated at 25 °C and maintained in Leibovitz’s medium (L-15; Gibco, Waltham, MA, USA), supplemented with 10% fetal bovine serum (FBS; Gibco, Waltham, MA, USA) and 1× antibiotic-antimycotic (AA; Sigma-Aldrich, St. Louis, MI, USA), producing a final concentration of 100 units/mL penicillin, 100 µg/mL streptomycin, and 0.25 µg/mL amphotericin B. A skin sample that tested positive for LMBV by using PCR and was confirmed by using Sanger sequencing was thawed, weighed, and diluted 1:25 in L-15 medium prior to homogenization using a Stomacher^®^ 80 Biomaster (Seward, Bohemia, NY, USA) for 2 min at the highest speed. After being transferred to a clean conical tube, the homogenate was clarified by centrifuging at 4 °C at 3000× *g* for 10 min. The supernatant was then transferred to another clean conical tube and diluted by adding an equal amount of L-15 containing 2× AA solution. The sample was allowed to incubate overnight at 4 °C prior to repeated clarification as described above and filtration using a 0.45 µm sterile syringe filter (Fisherbrand, Waltham, MA, USA). Following this, 1 mL of the supernatant was inoculated onto a confluent monolayer of EPC cells in a 25 cm^2^ culture flask. A separate 25 cm^2^ culture flask with an EPC monolayer was inoculated with 1 mL of L-15 media containing 2% FBS and 1× AA as a negative control. The flasks were gently rocked every 15 min for one hour, after which the supernatant was removed and 5 mL of L-15 media containing 2% FBS and 1× AA was added to each flask. The cells were observed daily for cytopathic effects (CPE). After CPE was observed in more than 90% of the infected cells, the remaining cells and spent media were collected and clarified by centrifugation at 4 °C at 3000× *g* for 10 min. The DNA from the clarified supernatant was extracted by using the DNeasy Blood and Tissue kit (Qiagen, Hilden, Germany) according to the manufacturer’s instructions and stored at −80 °C prior to whole-genome sequencing.

### 2.5. Whole-Genome Sequencing and Phylogenetic and Genetic Analyses

A DNA library was generated using a Nextera XT DNA kit (Illumina, San Diego, CA, USA) and sequenced on a MiSeq sequencer using a v3 600 cycle kit (Illumina, San Diego, CA, USA). Cell culture host sequences were removed from the sequencing data using the common carp genome (*Cyprinus carpio*; GenBank accession number GCA_000951615.2) as the reference genome using Kraken v2.0 (Johns Hopkins University School of Medicine, Baltimore, MD, USA) [23]. *De novo* assembly of the remaining paired-end reads was performed in SPAdes v3.15.3 [24] using the default settings. The quality of assembly was assessed by mapping reads back to the consensus sequence and visually inspecting alignments in CLC Genomics Workbench v20.0.4 using a window size of 1 base pair (bp). The assembled genome was then annotated using GenemarkS [25]. Additional open reading frames (ORF) were predicted using CLC Genomics Workbench v20.0.4 and by comparison to other LMBV genomes (GenBank accession numbers: MK681855 and MK681856). Acceptance of ORFs was based on the following criteria: (i) only ORFs longer than 120 nucleotides would be accepted; (ii) more than 25% overlap between neighboring ORFs was not accepted; and (iii) if overlap did occur, only the larger ORF was accepted unless ortholog ranavirus ORFs exist [26]. The functions of the ORFs were predicted based on BLASTP searches against the National Center for Biotechnology Information (NCBI) GenBank nonredundant protein sequence database.

The amino acid sequences of the 21 iridovirus core genes were retrieved from 27 ranaviruses available in the NCBI GenBank database and from LMBV sequenced in the current study (isolate WVL21117) (Table S1) [27]. They were then individually aligned in Geneious Prime v2019.2.3 by utilizing multiple alignment using the Fast Fourier Transformation (MAFFT) [28] option, followed by concatenation. A maximum likelihood (ML) analysis was performed using IQ-TREE v1.6.12 (http://iqtree.cibiv.univie.ac.at/; accessed on 27 December 2023) with 1000 bootstrap replicates to determine clade support. A separate ML analysis was performed based on the nucleotide alignment of the MCP gene to determine the relationship of LMBV isolate WVL21117 to DFV, GV6, and 15 additional LMBV isolates (Table S1), for which only the MCP gene was available in NCBI GenBank. A pairwise genetic distance analysis was also performed on the same MCP gene sequences using the Sequence Demarcation Tool v1.2 [29] with the MAFFT alignment option implemented.

## 3. Results

### 3.1. Gross Pathology and Bacteriology

**Figure 1:**
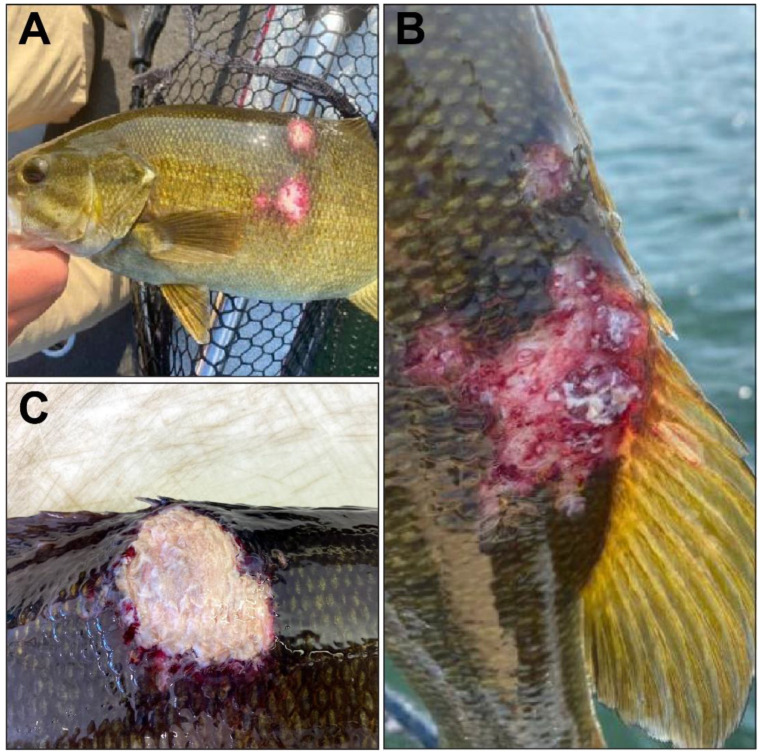
Gross observations of the ulcerative skin lesions on smallmouth bass (SMB; *Micropterus dolomieu)* included in this study. (**A**) SMB was observed with ulcers on the dorsal and lateral aspects of the body. (**B**) SMB was observed with a multifocal erosive to ulcerative lesion at the base of the dorsal fin. (**C**) SMB was observed with an ulcerative lesion at the base of the dorsal fin.

Necropsies were performed on 10 of the 14 SMB. Focally extensive ulcerative skin lesions of variable size were observed on the dorsal and/or lateral aspect of all fish, commonly at the base of the dorsal fin. These lesions were characterized by centrally necrotic tissue surrounded by a margin of inflamed tissue (Figure 1). Wet mounts of skin and gills were unremarkable. Upon gross internal examination, ecchymotic hemorrhage of the swim bladder was noted in two fish, and all 10 livers ranged from tan to orange in color. Other gross findings included masses (~2 cm × 2 cm) on the posterior kidney of two fish, extensive coelomic adhesions in one animal, and bilateral corneal opacification noted in another. The examination of the remaining organ systems produced no significant findings. Three Gram-negative bacterial species were isolated from the skin samples: *Flavobacterium columnare* (*n* = 2; HS plate at 22 °C), a *Chryseobacterium* sp. (*n* = 1; HS plate at 22 °C), and a motile *Aeromonas* sp. (*n* = 4; TSA plate at 22 °C). *Flavobacterium columnare* was identified and confirmed by using the Griffin screening method [30]. The other bacterial species were identified using matrix-assisted laser desorption ionization–time-of-flight mass spectrometry (MALDI-TOF MS, Bruker Daltonics, Bellerica, MA, USA). No bacterial growth was observed on the 15 °C HS plate for any sample. Similarly, none of the kidney inoculates generated bacterial growth. 

### 3.2. Histopathology

**Figure 2:**
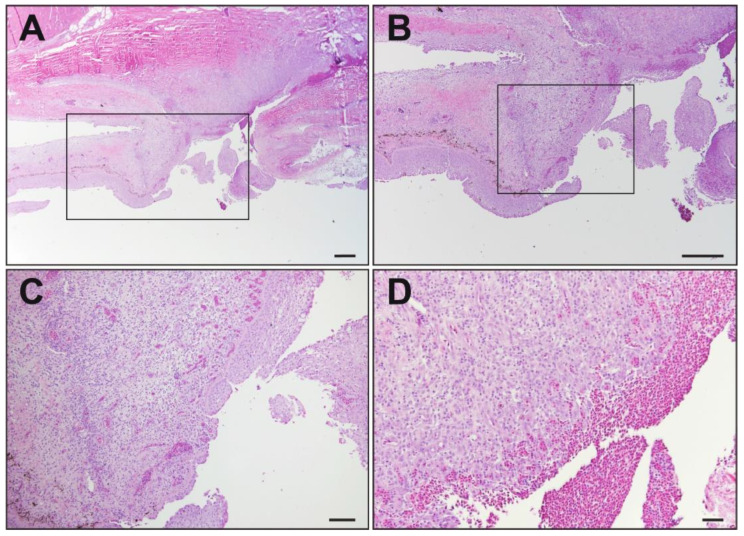
Histological sections of ulcerated skin of smallmouth bass. (**A**) Ulcerated section of skin from fish: discontinuous epidermis; inflammation that extends through the dermis and into the underlying skeletal muscle. H&E. Scale bar = 200 μm. (**B**) Increased magnification of black box in image (**A**). H&E. Scale bar = 200 μm. (**C**) Increased magnification of black box in Image (**B**). H&E. Scale bar = 50 μm. (**D**) Section of extensive ulceration, lack of epidermis and replacement by red blood cells (hemorrhage), underlying dermis infiltrated by granulation tissue, and mononuclear inflammation. H&E. Scale bar = 20 μm.

Microscopic evaluation of the kidney, liver, spleen, and swim bladder from all 10 fish was unremarkable. All the fish had similar ulcerative skin lesions that extended from the epidermis and superficial dermis into the deep dermis and underlying skeletal muscle. All the skin lesions were characterized by infiltration of the mononuclear inflammatory cells as well as areas of hemorrhage (Figure 2). Mixed bacteria were present in all examined skin lesions. In seven of the fish, there was evidence of dermal fibrosis suggestive of chronic/previous injury. There was no evidence of coelomitis or systemic infection.

### 3.3. PCR Amplification and Virus Isolation

**Figure 3:**
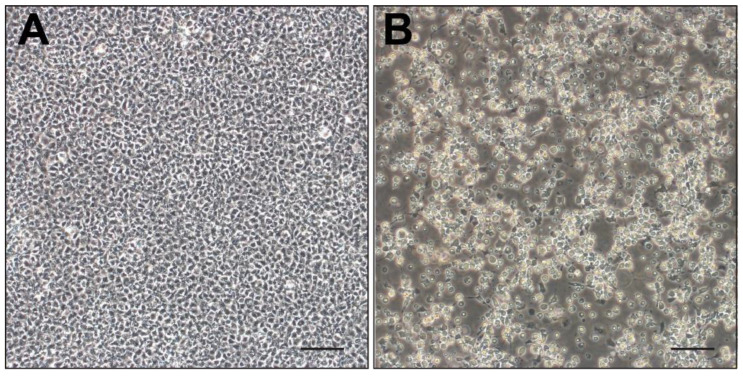
Epithelioma papulosum cyprini cells inoculated with skin lesion homogenate; day 5 post-infection. (**A**) Uninfected control. (**B**) Cytopathic effects characterized by enlarged, refractile, and lysed cells detaching from the monolayer. Scale bar = 100 μm.

All 14 skin samples tested positive for LMBV using conventional PCR. In contrast, only eight weakly positive (lighter bands) results for LMBV were generated from the pooled internal organ samples from the same 14 fish. The skin sample (WVL21117-2D) with the brightest band on PCR was confirmed as LMBV by using Sanger sequencing, and it was chosen for virus isolation. The infected EPC cells began to show CPE 24 h post-inoculation, characterized by enlarged, refractile, and lysed cells detaching from the monolayer. Considerable CPE was observed by day 6, resulting in the destruction of more than 90% of the monolayer (Figure 3). No CPE was observed on the control EPC cells at any point throughout the 14 days.

### 3.4. Whole-Genome Assembly and Phylogenetic and Genetic Analyses

The next-generation sequencing generated 3,774,741 reads, of which 74.4% were classified as the cell culture host genome and removed by using Kraken v2.0. The de novo assembly of the remaining paired-end reads (25.6%) by SPAdes v3.15.3 resulted in a 99,328 bp LMBV genome with a 51.9% G + C content. The LMBV isolate WVL21117 was predicted to encode 100 ORFs (Table S2). Comparative genomic analysis of the isolate WVL21117 to two LMBV genomes reported from the United States (isolates Pine 14-204 and Alleghany 12-343) revealed that 13 ORFs in the Pine 14-204 isolate and 14 ORFs in the Alleghany 12-343 isolate were not previously annotated (Table S2). An ORF (LMBV_38) identified within the Pine 14-204 and Alleghany 12-343 isolates was not included in the WVL21117 genome annotation due to greater than 50% overlap with the neighboring ORF, and no homologous sequence could be identified in other ranaviruses. A region within ORF 64 (hypothetical protein) of the isolate WVL21117 was scrutinized further by using PCR and Sanger sequencing. It was found to span 1725 bp in the isolate WVL21117, compared to 1683 bp in Alleghany 12-342 and 411 bp in Pine 14-204. Additional evaluation revealed that this ORF had been annotated as two separate genes within the Pine 14-204 genome (LMBV_66 and 67) due to two frameshift mutations caused by insertions of thymine and cytosine at positions 71,064 and 71,133, respectively. The nucleotide sequences of the 21 iridovirus core genes were found to be identical between the three isolates. Further comparative analysis of the remaining genes revealed nearly identical genomes, in which 7 ORFs ranged between 54.3 and 98% identity, 19 ORFs were 99.1–99.9% identical, and the remaining 53 ORFs were indistinguishable. Furthermore, out of 100 ORFs, 12 are unique to *Santee-Cooper ranavirus* and were not detected in any other virus (Table S2).

**Figure 4:**
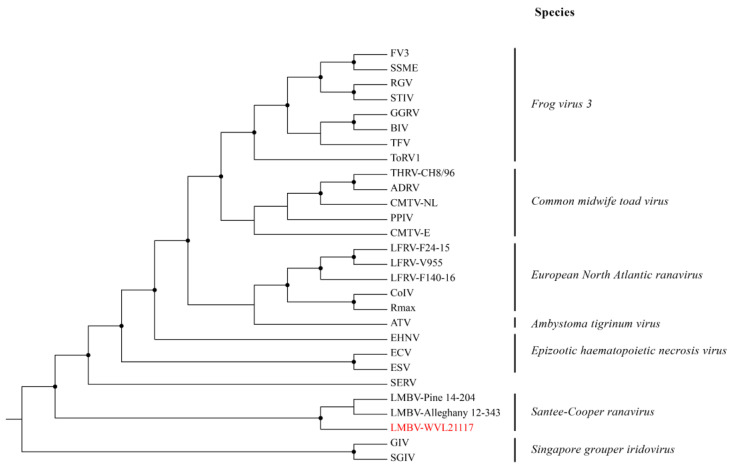
Maximum likelihood cladogram depicting the relationship of the largemouth bass virus isolate WVL21117 (highlighted in red) to 27 other ranaviruses based on the concatenated amino acid sequence alignments of 21 iridovirus core genes. Nodes with black circles are supported by bootstrap values ≥ 80%. See Table S1 for taxa abbreviations.

**Figure 5:**
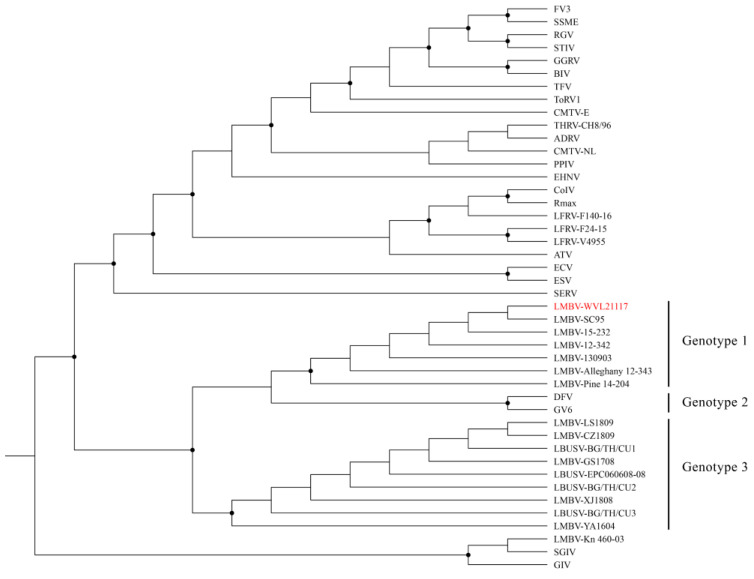
Maximum likelihood cladogram depicting the relationship of the largemouth bass virus isolate WVL21117 (highlighted in red) to 43 other ranaviruses based on the nucleotide sequence alignment of major capsid protein. Nodes with black circles are supported by bootstrap values ≥ 80%. See Table S1 for taxa abbreviations.

The ML analysis based on the concatenated amino acid alignments of 21 iridovirus core genes generated a well-supported tree and grouped the three LMBV isolates (WVL21117, Pine 14-204, and Alleghany 12-343) within the genus *Ranavirus* and species *Santee-Cooper ranavirus* (Figure 4). The ML analysis based on the MCP gene alignment generated a tree with a similar topology to the tree built based on the 21 iridovirus core genes (Figure 5). Additionally, pairwise nucleotide comparison of the MCP gene showed that the LMBV isolate WVL21117 is identical to the LMBV isolates from the United States (Pine 14-204, Alleghany 12-343, 12-342, 15-232, SC95, and 130903). The aforementioned strains are nearly identical to DFV and GV6 (99.2%), as well as to the LMBV isolates from Thailand (99.1%; BG/TH/CU1, BG/TH/CU2, and BG/TH/CU3) and China (99.1%; LS1809, XJ1808, CZ1809, YA1604, EPC060608-08, and GS1708) (Figure S1). In contrast, the LMBV isolate Kn 460-03 is grouped within the species *Singapore grouper iridovirus* (SGIV) clade with high bootstrap support. Furthermore, isolate Kn 460-03’s MCP gene is 99.4% identical to SGIV and only 71.1% identical to WVL21117.

## 4. Discussion

In this study, we report the detection, isolation, and genomic characterization of LMBV from wild SMB. The common pathologic features previously reported in LMBV-infected fish include coelomitis, exophthalmia, organomegaly, necrosis of internal organs, over-inflation of the swim bladder, and ulcerative skin lesions [13,14]. In the current study, the most consistent clinical sign identified in the LMBV-infected SMB was ulcerative skin lesions. Further sampling of affected wild fish or experimental challenge studies are required to determine the potential relationship of LMBV to the remaining clinical signs, such as coelomic adhesions, corneal opacification, ecchymotic hemorrhage of the swim bladder, kidney masses, and liver discoloration.

The phylogenetic analysis based on the 21 core iridovirus genes supported the LMBV isolated from SMB as a member of the species *Santee-Cooper ranavirus* within the genus *Ranavirus*. In addition, the LMBV isolate WVL21117 contains a G + C content within the reported range (49–55%) and encodes the cytosine methyltransferase gene (ORF23), typical of most ranaviruses [3]. Comparative genomic analysis resulted in the detection of 12 genes exclusive to LMBV isolates, including WVL21117, Pine 14-204, and Alleghany 12-343. This demonstrates that *Santee-Cooper ranavirus* is, in fact, a separate species, as first suggested based on MCP and DNA methyltransferase genes analyses [2,4]. In addition, the *Santee-Cooper ranavirus* forms one of the basal branches of the genus surrounded by other fish pathogens, such as *Singapore grouper iridovirus* and *Epizootic haematopoietic necrosis virus*.

The ML and genetic analyses based on the MCP gene sequence alignments suggest regional variation between isolates of the species *Santee-Cooper ranavirus*. Three subclades are formed within the species, possibly representing three different genotypes. The first subclade includes isolates from the United States, representing the first genotype. Within this subclade are the following isolates with identical MCP gene sequences: WVL21117, Pine 14-204, Alleghany 12-343, 12-342, 15-232, SC95, and 130903. Only isolate WVL21117 was found in SMB, while the remaining isolates were found in LMB. The second subclade includes isolates from Southeast Asia (DFV and GV6), representing genotype two. The same pattern (i.e., identical MCP gene sequences) was observed for DFV and GV6. The third subclade includes isolates from China and Thailand that represent genotype three. Within this subclade are the following isolates with identical MCP gene sequences: LS1809, XJ1808, CZ1809, YA1604, GS1708, EPC060608-08, BG/TH/CU1, BG/TH/CU2, and BG/TH/CU3.

It is important to note that although MCP gene sequences of regional isolates are identical, this does not indicate that a single virus is circulating in each region. This is partially demonstrated by the identical MCP gene sequences of isolates WVL21117, Pine 14-204, and Alleghany 12-343, despite differing genome lengths. Variations in sequence length could represent sequencing artifacts, variability in the size of repeat regions, or changes reflecting adaptation to different hosts. Nonetheless, full genome or multiple gene analyses are required to determine the genetic diversity or variation in LMBV isolates from different regions.

The LMBV isolate Kn 460-03 from Taiwan showed 70.8–71.2% identity to LMBV isolates from China, Southeast Asia, Thailand, and the United States. In contrast, isolate Kn 460-03’s MCP gene sequence is closely related to established isolates within the species *Singapore grouper iridovirus*, and is 99.2% identical to SGIV and 98.7% identical to grouper iridovirus. The ML phylogenetic analysis based on the MCP gene also supports the grouping of isolate Kn 460-03 within the species *Singapore grouper iridovirus* clade with high bootstrap value. Therefore, isolate Kn 460-03 is likely an isolate of *Singapore grouper iridovirus*.

It has been demonstrated experimentally that water temperature plays a role in the pathogenicity of LMBV [14]. Smallmouth bass exposed to LMBV at 28 °C experienced 50% mortality, whereas SMB exposed to LMBV at 23 °C and 11 °C experienced 10% and no mortality, respectively [14]. The analysis of localized endemics has also supported a heavy environmental influence on virulence. For example, the Sardis Reservoir in Mississippi experienced a mass mortality event of at least 3000 LMB in September 1998 [17]. During this time, there was significant fishing pressure, elevated water temperatures ranging from 29 to 32 °C, and evidence of destratification leading to low dissolved oxygen. Surveys conducted in the following years found LMBV to be present, but the clinical disease was less severe. In the current study, environmental water temperature was not obtained, but diseased fish were collected during early fall. The water temperature may have been lower than the optimal temperature (i.e., 28 °C) for the replication of LMBV, which may explain why mortality was not observed. The previously mentioned study [14] also found that co-infection of LMBV and *Flavobacterium columnare* or *Aeromonas salmonicida* increased mortality in SMB. In the current study, *F. columnare* was isolated from two SMB and an *Aeromonas* sp. was isolated from four SMB. Variations in virulence of co-infecting bacteria and lower water temperature may have played a role and resulted in less severe disease. Furthermore, an LMBV mortality event at the time of SMB collection could have gone unnoticed due to the large size of Green Bay. Nevertheless, the pathology observed in the wild fish in the current study compared with findings from the previous study [14] suggests a relationship between LMBV pathogenicity and water temperature. Due to the multifactorial nature of LMBV, a full set of water parameters should be taken during future outbreaks to elucidate the possible relationship of environmental influence on pathogenicity. Additional challenge studies will also be necessary to thoroughly comprehend the affiliation of biotic and abiotic factors influencing infection.

Although the pathogenesis of LMBV is not well studied, our findings suggest that wild fish are infected through the water column, with the skin being the likely portal of entry. All 14 skin samples tested positive for LMBV by using conventional PCR, while only 8 internal organ samples produced weakly positive results. Furthermore, all necropsied SMB were found to have ulcerative skin lesions while lacking histological evidence of systemic disease. In a 2018 study, ulcerative skin lesions were unique to SMB infected with LMBV through immersion [14]. It appears that LMBV has a predilection for skin; however, continued research is required to fully understand the pathogenesis of LMBV.

As previously stated, SMB are important aquatic predators that control prey population dynamics in shallow, warm waters. This environmental preference has the potential to make them more vulnerable to LMBV. Continued surveillance efforts will cultivate a deeper understanding of the true prevalence and current persistence of this virus in both wild and managed populations. The monitoring of LMBV in hatcheries will also prevent the introduction of this virus into naïve bodies of water during restocking efforts. In addition, virus isolation and complete genomic sequencing of future isolates are required to fully understand the evolutionary relationship between isolates of the species *Santee-Cooper ranavirus* and the origin and global temporospatial spread of this virus.

## Data Availability

The complete genome sequence of the largemouth bass virus isolate WVL21117 has been deposited in GenBank under accession numbers PP526145.

## Supplementary Materials

The following supporting information can be downloaded at: https://www.mdpi.com/article/10.3390/v16050715/s1, Figure S1: Genetic comparison of the nucleotide sequences of the major capsid protein; Table S1: Details of ranaviruses used in the phylogenetic and genetic analyses; Table S2: Summary of the genome annotations.
