# Supplementary material for: Phylogenomic Characterization of Ranavirus Isolated from Wild Smallmouth Bass (Micropterus dolomieu)
Source: Viruses. 2024 Apr 30;16(5):715. doi: 10.3390/v16050715 (PMC11126109; doi:10.3390/v16050715)
Supplement: Supplementary file 1 [file viruses-16-00715-s001.zip › viruses-2961598-supplementary.pdf]

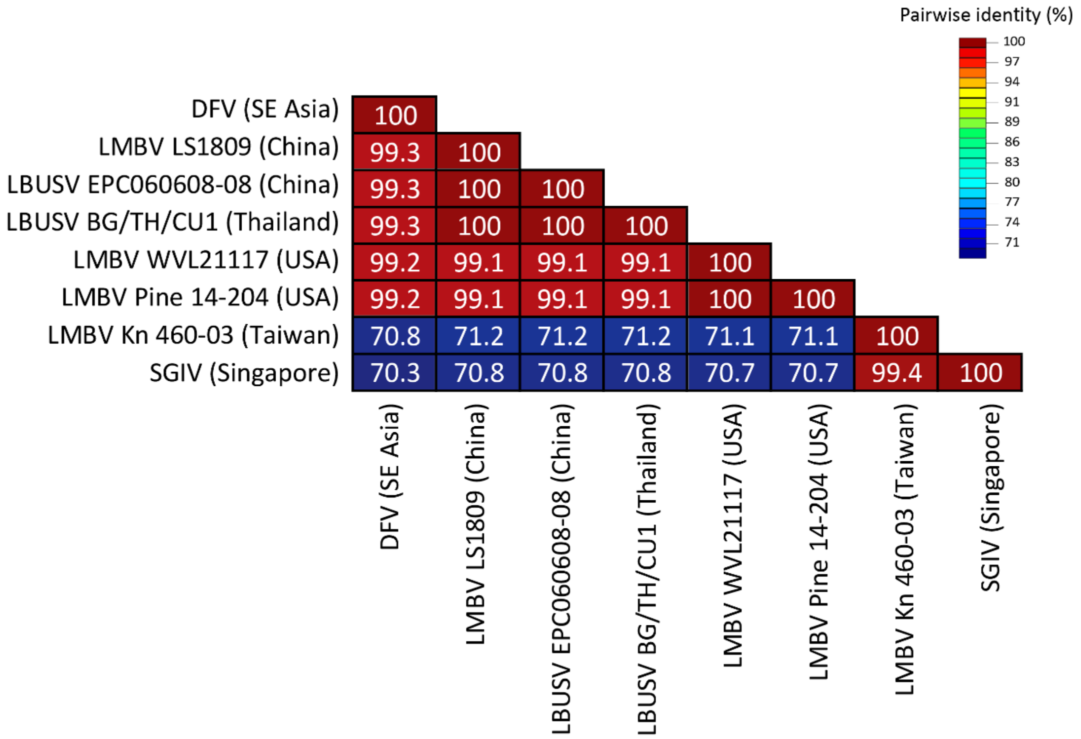

Figure S1 Genetic comparison of the nucleotide sequence of the major capsid protein of largemouth bass virus (LMBV) isolate WVL21117 to representative LMBV isolates and Singapore grouper iridovirus. LMBV isolates Alleghany 12-343, 12-342, 15-232, SC95, 130903 were identical to LMBV-WVL21117, LMBV isolates BG/TH/CU2, BG/TH/CU3, XJ1808, CZ1809, YA1604, GS1708 were identical to EPC060608-08, and guppy virus 6 is identical to doctor fish virus; hence, they were excluded from the genetic analysis. Values are expressed as a percentage of nucleotide identity. See Table S1 for virus abbreviations.

Table S1. Virus species, isolates names, abbreviations, and GenBank accession numbers of the ranaviruses used in the phylogenetic and genetic analyses.

Virus species	Isolate name (abbreviation)	GenBank accession number
Frog virus 3	Frog virus 3 (FV3)	AY548484
	Frog virus 3 isolate SSME (SSME)	KJ175144
	Soft-shelled turtle iridovirus (STIV)	EU627010
	Rana grylio iridovirus (RGV)	JQ654586
	Tiger frog virus (TFV)	AF389451
	Bohle iridovirus (BIV)	KX185156
	German gecko ranavirus (GGRV)	KP266742
	Tortoise ranavirus isolate (ToRV1)	KP266743
Common midwife toad virus	Common midwife toad virus (CMTV-E)	JQ231222
	Common midwife toad virus (CMTV-NL)	KP056312
	Testudo hermanni ranavirus (THRV-CH8/96)	KP266741
	Andrias davidianus ranavirus (ADRV)	KC865735
	Pike-perch iridovirus (PPIV)	KX574341
European North Atlantic ranavirus	Lumpfish ranavirus isolate F24-15 (LMRV-F24-15)	MH665358
	Lumpfish ranavirus isolate F140-16 (LMRV-F140-16)	MH665359
	Lumpfish ranavirus isolate V4955 (LMRV-V4955)	MH665360
	Ranavirus maximus (Rmax)	KX574343
	Cod iridovirus (CoIV)	KX574342
Ambystoma tigrinum virus	Ambystoma tigrinum virus (ATV)	AY150217
Epizootic haematopoietic necrosis virus	Epizootic haematopoietic necrosis virus (EHNV)	FJ433873
	European catfish virus (ECV)	KT989885
	European sheatfish virus (ESV)	JQ724856
Santee-Cooper ranavirus	Largemouth bass virus isolate WVL21117 (LMBV-WVL21117)	
	Largemouth bass virus isolate Alleghany 12-343 (LMBV-Alleghany 12-343)	MK681855
	Largemouth bass virus isolate Pine 14-204 (LMBV-Pine 14-204)	MK681856
	Largemouth bass virus isolate 12-342 (LMBV-12-342)*	KY825779
	Largemouth bass virus isolate 15-232 (LMBV-15-232)*	KY825781
	Largemouth bass virus isolate 130903 (LMBV-130903)*	KY825782
	Largemouth bass virus isolate Santee-Cooper Reservoir (LMBV- SC95)*	FR682503

	Largemouth bass virus isolate LS1809 (LMBV-LS1809)*	MK836315
	Largemouth bass virus isolate CZ1809 (LMBV-CZ1809)*	MK836316
	Largemouth bass virus isolate XJ1808 (LMBV-XJ1808)*	MK836317
	Largemouth bass virus isolate GS1708 (LMBV-GS1708)*	MK836318
	Largemouth bass virus isolate YA1604 (LMBV-YA1604)*	MK836319
	Largemouth bass ulcerative syndrome virus isolate EPC060608-08 (LBUSV- EPC060608-08)*	GU256635
	Santee-Cooper ranavirus isolate BG/TH/CU1(LBUSV-BG/TH/CU1)*	KU507315
	Santee-Cooper ranavirus isolate BG/TH/CU1(LBUSV-BG/TH/CU2)*	KU507316
	Santee-Cooper ranavirus isolate BG/TH/CU1(LBUSV-BG/TH/CU3)*	KU507317
	Doctorfish virus (DFV)*	FR677324
	Guppy virus 6 (GV6)*	FR677325
Singapore grouper iridovirus	Singapore grouper iridovirus (SGIV)	AY521625
	Grouper iridovirus (GIV)	AY666015
	Largemouth bass virus isolate Kn 460-03 (LMBV-Kn 460-03)*	JF264364
Unclassified	Short-finned eel ranavirus (SERV)	KX353311

*Used only in MCP gene analysis

Table S2. Summary of the genome annotations of the largemouth bass virus isolate WVL21117.

ORF	Position in the genome	Product size (amino acid)	Predicted function
1#	1-762	253	Replication factor
2#	807-1700	297	Hypothetical protein
3	1747-1959	70	Hypothetical protein
4#	1992-4859	955	D5 family NTPase/ATPase
5*	4981-6672	563	Hypothetical protein
6#	6697-7368	223	Hypothetical protein
7*	7423-7956	177	Hypothetical protein
8	7956-8729	257	Hypothetical protein
9	8737-9459	240	Hypothetical protein
10	9492-10013	173	Hypothetical protein
11	10058-10534	158	Hypothetical protein
12	10633-11379	248	Hypothetical protein
13	11499-11978	159	Hypothetical protein
14	11989-14862	957	Tyrosine kinase
15	14952-15764	270	eIF-2 alpha-like protein
16*	15808-16113	101	Hypothetical protein
17	16195-17589	464	ATPase-dependent protease
18#	17633-18790	385	Ribonuclease III
19#	18820-19095	91	Transcription elongation factor SII
20	19158-19601	147	Immediate early protein ICP-18
21	19662-20252	196	Hypothetical protein
22	20358-20936	192	Hypothetical protein
23	20921-21583	220	Cytosine DNA methyltransferase
24	21649-22224	191	Tumor necrosis factor receptor
25#	22304-23815	503	Myristylated membrane protein
26*	23839-24012	57	Hypothetical protein
27	24084-25148	354	3-beta-hydroxy-delta 5-C27 steroid oxidoreductase-like protein
28	25217-26341	374	Hypothetical protein
29	26461-27249	262	Hypothetical protein
30*	27719-28027	102	Hypothetical protein
31	28359-28808	149	Hypothetical protein
32	28828-29187	119	Hypothetical protein
33	29290-29478	62	Hypothetical protein
34	29532-31397	621	Neurofilament triplet H1-like protein
35	31412-31837	141	Hypothetical protein
36*	31878-32042	54	Hypothetical protein

37*	32079-32840	253	Hypothetical protein
38	32867-34081	404	Hypothetical protein
39	34108-34980	290	Hypothetical protein
40#	34986-36050	354	Myristylated membrane protein
41#	36134-37282	382	Immediate early protein ICP-46
42#	37395-38786	463	Major capsid protein
43	38900-40072	390	Hypothetical protein
44#	40078-40521	147	Thiol oxidoreductase
45	40555-42339	594	Hypothetical protein
46	42422-43150	242	Proliferating cell nuclear antigen
47	43202-43771	189	Deoxynucleoside kinase
48	43841-44239	132	Hypothetical protein
49#	44261-47110	949	NTPase
50#	47128-47745	205	Putative NIF/NLI interacting factor
51	47785-48024	79	Hypothetical protein
52	48116-49804	562	Ribonucleotide reductase alpha subunit
53*	49868-50188	106	Hypothetical protein
54	50247-50522	91	Hypothetical protein
55#	50576-54064	1162	Hypothetical protein
56	54134-54493	119	Hypothetical protein
57#	54460-55407	315	AAA-ATPase
58	55620-56465	281	Hypothetical protein
59	56549-58060	503	Hypothetical protein
60	58093-58434	113	Hypothetical protein
61	58136-60775	879	Serine/theronine protein kinase
62	60803-61237	144	Hypothetical protein
63	61288-62988	566	Hypothetical protein
64*	63061-64785	574	Hypothetical protein
65*	64826-65047	73	Hypothetical protein
66	65087-65497	136	Hypothetical protein
67*	65531-66232	233	Neurofilament triplet H1-like protein
68	66278-66688	136	Hypothetical protein
69	66743-67546	267	Hypothetical protein
70#	68083-68547	154	Hypothetical protein
71#	68616-69686	356	Putative DNA repair protein RAD2
72#	69730-73014	1094	DNA-dependent RNA Polymerase II beta subunit
73	73078-73614	178	Hypothetical protein
74	73694-74407	237	Tumor necrosis factor receptor
75	74507-74932	141	dUTPase
76	74995-75270	91	Caspase recruitment domain protein

77	75388-76551	387	Ribonucleoside reductase beta subunit
78	76600-77121	173	Hypothetical protein
79	77143-77859	238	Hypothetical protein
80#	77919-80933	1004	DNA polymerase
81	80993-81541	182	Hypothetical protein
82	81608-82723	371	Hypothetical protein
83	82790-83416	208	Hypothetical protein
84	83466-83687	73	Hypothetical protein
85	83746-84003	85	Putative LITAF/PIG7 possible membrane-associated motif in LPS-induced Tumor Necrosis Factor Alpha factor
86	84052-85185	377	Hypothetical protein
87	85212-86429	405	Hypothetical protein
88	86549-87523	324	NTPase/helicase-like protein
89	87620-88270	216	Hypothetical protein
90	88357-88596	79	Hypothetical protein
91	88604-88975	123	Hypothetical protein
92*	89026-89226	66	Hypothetical protein
93	89400-89921	173	Hypothetical protein
94#	89981-91459	492	Phosphotransferase
95	91468-91878	136	Hypothetical protein
96	92073-93278	401	Helicase-like Protein
97	92925-93335	136	Hypothetical protein
98#	93595-97659	1354	DNA-dependent RNA Polymerase II alpha subunit
99	98073-98753	226	Hypothetical protein
100	98756-99271	171	Bcl-2 family apoptosis regulator protein

*ORFs unique to *Santee-Cooper ranavirus*
#iridovirus core genes
